# Unusual [^18^F]FDOPA PET-CT maging of insulinomas in children

**DOI:** 10.1186/s41824-026-00331-6

**Published:** 2026-10-28

**Authors:** Sameera Hannah Auckburally, Ross J. Craigie, Konrad Mohnike, Sabrina Alam, Sarah Worthington, Indraneel Banerjee, Rakesh Sajjan

**Affiliations:** 1 Department of Paediatric Endocrinology, Royal Manchester Children’s Hospital, Manchester, UK; 2 Department of Paediatric Surgery, Royal Manchester Children’s Hospital, Manchester, UK; 3 Diagnostic and Therapeutic Center, Frankfurter Tor (DTZ), Berlin, Germany; 4 Department of Paediatric Radiology, Royal Manchester Children’s Hospital, Manchester, UK; 5 Faculty of Biology, Medicine and Health, University of Manchester, Manchester, UK; 6 Department of Nuclear Medicine, Royal Manchester Children’s Hospital, Manchester, UK

**Keywords:** Insulinoma, [^18^F]FDOPA, Paediatrics, Endocrinology

## Abstract

**Purpose:**

Insulinomas are rare pancreatic neuroendocrine tumours, causing recurrent episodes of hypoglycaemia. Accurate localisation is essential for surgical management, yet imaging remains challenging. This case series presents unusual findings of paediatric insulinomas on [^18^F]-dihydroxyphenylalanine ([^18^F]FDOPA) positron emission tomography-computed tomography (PET-CT) imaging.

**Methods:**

We retrospectively reviewed medical notes and investigations of histologically proven paediatric insulinoma cases (n=4) presenting to Royal Manchester Children’s Hospital between 2008 and 2024. All patients underwent [^18^F]FDOPA PET-CT as part of their diagnostic workup. Imaging findings were analysed, with particular attention to tracer uptake patterns.

**Results:**

Contrary to the increased tracer uptake typically seen in neuroendocrine tumours, three of the four insulinomas demonstrated focal photopenia at the site of the tumour on [^18^F]FDOPA PET-CT. Only one case showed increased uptake, suggesting a predominance of reduced, rather than enhanced, tracer activity in paediatric insulinomas

**Conclusion:**

Photopenia on [^18^F]FDOPA PET-CT may be a more frequent finding than increased uptake in paediatric insulinomas. Recognizing this atypical pattern is important for accurate tumour localisation before surgical management. Further studies are needed to validate these findings and find a mechanistic explanation.

## Introduction

Insulinomas are rare pancreatic neuroendocrine tumours arising from the pancreatic islet beta cells, with an incidence of 1–4 per million per year (Herder et al. 2023, Melikyan et al. 2023). Insulinomas in the paediatric population are even more uncommon, accounting for 1% of all insulinomas. Hence, literature on paediatric insulinomas is scarce.

Ninety percent of insulinomas are benign, but malignancy may occur in 5–10%. The majority of insulinomas are solitary lesions; approximately 10% present as multiple lesions, either concurrently or sequentially over time (Murray et al. 2025). Clinical presentation is generally recurrent episodes of hypoglycaemia, which may manifest as neuroglycopenic symptoms such as confusion, drowsiness, seizures, syncope and behavioural difficulties. However, given the non-specific symptoms and the rarity of insulinomas in children, in contrast to the more prevalent condition of congenital hyperinsulinism, diagnosis can be challenging (Melikyan et al. 2023, Miron et al. 2016).

Diagnosis of suspected cases is based on the detection of insulin at the time of hypoglycaemia, as in children with congenital hyperinsulinism (Banerjee et al. 2018); An undetectable insulin level does not always exclude an insulinoma (Okabayashi et al. 2013). As insulinoma cells produce relatively more proinsulin compared to normal pancreatic beta cells, proinsulin assays are sometimes used as a diagnostic adjunct (Okabayashi et al. 2013).

Those with insulinomas can be cured by successful surgical resection of the tumour; it is common practice, once an insulinoma is suspected biochemically, that preoperative localisation of the tumour is conducted with radiological and isotope imaging. Multiple non-invasive imaging modalities are available for the localisation of suspected insulinoma, including ultrasound, computed tomography (CT), magnetic resonance imaging (MRI) and somatostatin receptor scintigraphy (SRS), however these generally localise only 10–60% primary pancreatic neuroendocrine tumours (Modlin et al. 2008). Endoscopic ultrasound is considered to be the most sensitive modality for imaging small pancreatic neuroendocrine tumours (Lee et al. 2018). Neuroendocrine tumours have been found to have increased activity of L-DOPA decarboxylase which has allowed the use of [^18^F]-dihydroxyphenylalanine ([^18^F]FDOPA) to be used as a tracer to image insulinomas in positron emission tomography – computed tomography (PET-CT) scans (Kauhanen et al. 2010). Insulinomas are generally considered to be [^18^F]FDOPA avid with greater uptake and retention in tumour cells (Imperiale et al. 2015, Nakuz et al. 2018). In contrast to expectation, we report unusual photopenia in the tumour site in three of the four paediatric insulinomas presenting to our centre.

## Methods

On retrospective review of medical records, a total of four children were identified with a diagnosis of insulinoma between the years of 2008 and 2023. All received medical and surgical management at Royal Manchester Children’s Hospital. All underwent an [^18^F]FDOPA PET-CT scan as part of their diagnostic evaluation, three were undertaken at Royal Manchester Children’s Hospital, UK, and one in Sana Klinikum, Berlin, Germany. None of the children received carbidopa prior to imaging. 

### Compliance with ethical standards

Informed consent was obtained from the parents of all children included in the study, or from the participants themselves where they had reached the age of consent. Signed consent forms have been submitted to the journal editors. The authors declare that there are no conflicts of interest directly related to this manuscript.

### Case One

**Fig. 1:**
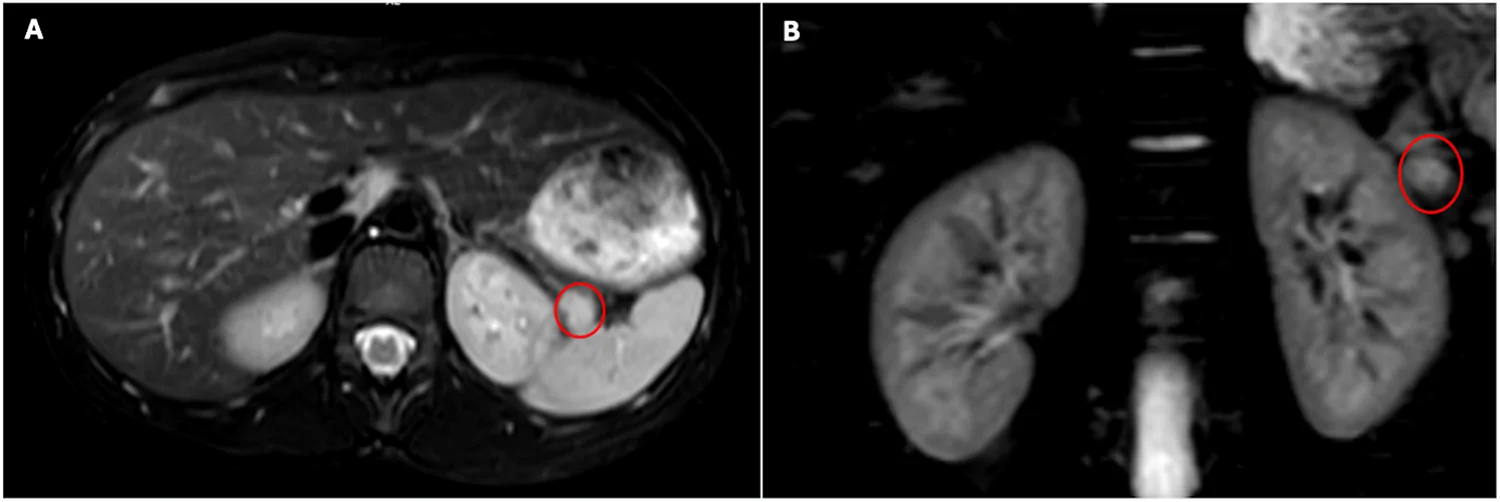
MR pancreas demonstrates hyperintense lesion in the pancreatic tail (red circle) on fat suppressed T2 weighted (SPIR) axial (**A**) and coronal (**B**) views. SPIR – Spectral presaturation with inversion recovery

A ten year old male initially presented to their local hospital with acute confusion associated with hypoglycaemia. He was further investigated by the regional paediatric endocrinology unit, started on diazoxide and found to have a tumour in the pancreatic tail on MR scan (Fig. 1). He was found to have a mutation in MEN1 on further testing.

**Fig. 2:**
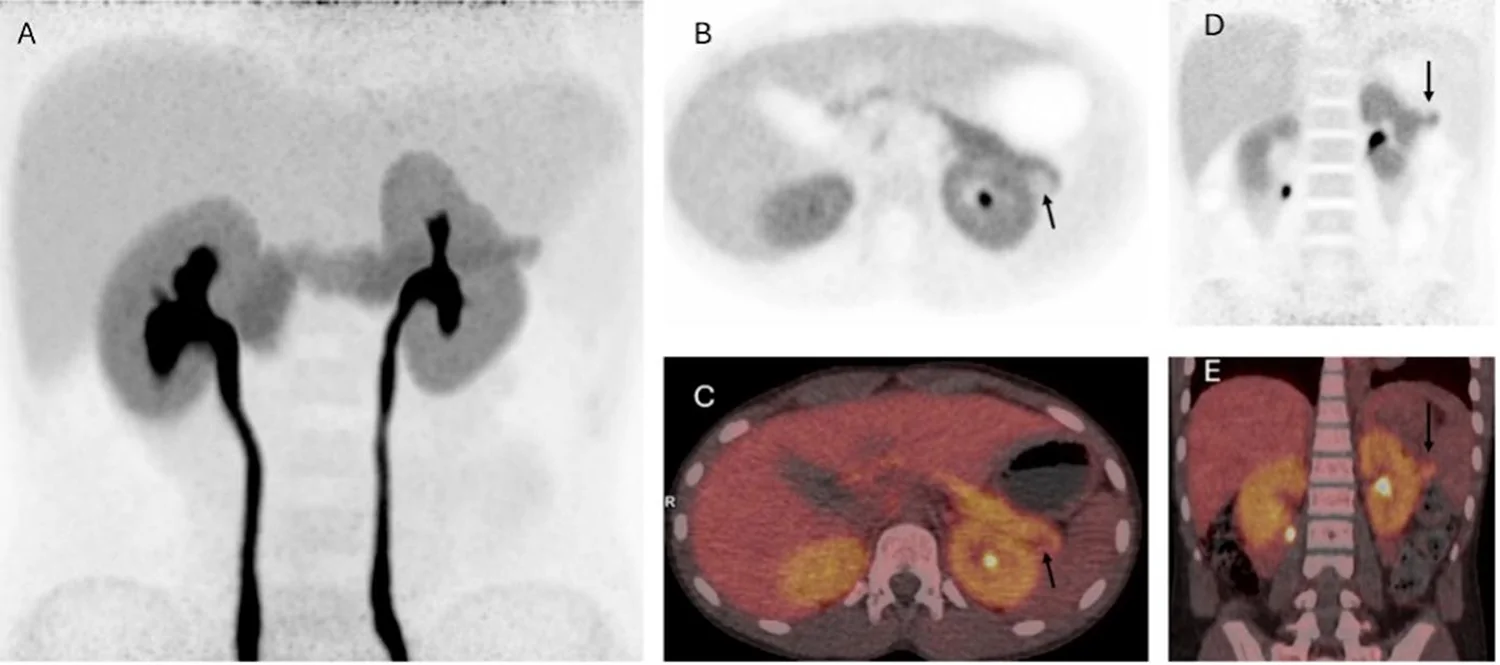
[^18^F]FDOPA PET CT images of Case One demonstrating area of photopenia in pancreatic tail corresponding to the lesion seen on MR imaging. (**A**) Maximum intensity projection (MIP) which does not show focal uptake. (**B**) and (**D**) – PET images axial and coronal projections respectively showing photopenic area in the tail of pancreas (black arrows). Figure (**C**) and (E) – Fused PET/CT images. Photopenia is better demonstrated on PET alone images in the top row

The patient was transferred to our centre for an [^18^F]FDOPA PET-CT scan (Fig. 2). Sequential images were obtained up to 90 minutes following injection of 4MBq/kg^18^F-FDOPA. The low dose CT component was acquired for the purposes of attenuation correction. A diagnostic CT with contrast was also conducted for image fusion. Arterial and venous phase diagnostic CT scans were acquired with intravenous contrast. An area of photopenia was observed in the tip of the tail of the pancreas corresponding to a lesion identified by MR imaging (13 mm x 10 mm),whilst the rest of the pancreas showed diffuse physiological uptake.

Due to the potential for multifocal disease in insulinomas related to multiple endocrine neoplasia (MEN) (Murray et al. 2025), the patient underwent an endoscopic ultrasound assessment of the pancreas to aid in clarifying the number of lesions. It demonstrated a 11 mm wide lesion consistent with previous imaging. This was resected laparoscopically achieving cure from hypoglycaemia. Histology demonstrated a well-differentiated insulin-producing neoplasm of the pancreas (Grade 1, pT2).

### Case Two

A 14 year old female initially presented to her local paediatric unit with a hypoglycaemic seizure after a three month history of short-lived episodes of confusion and unusual speech patterns. She had previously been fit and well with no developmental concerns. In terms of family history, her father had a stroke at the age of 34.

**Fig. 3:**
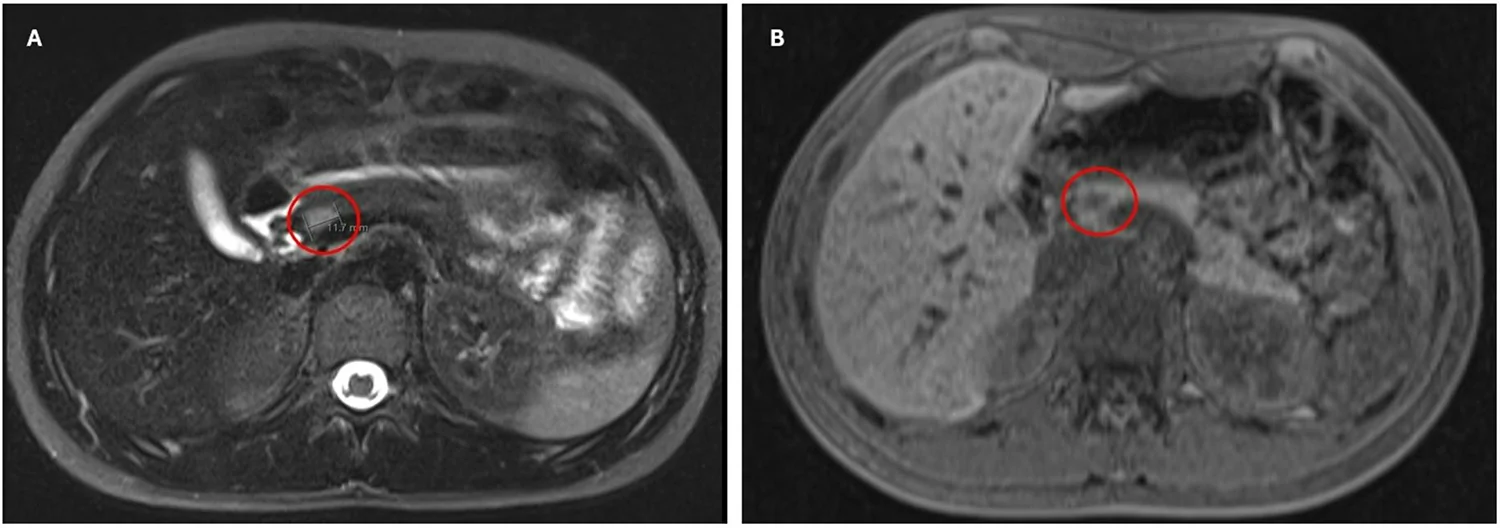
MR pancreas demonstrating lesion (red circle) in the pancreatic head anterior to the portal venous confluence. It is hyperintense on the axial view of the STIR (**A**) and hypointense on the axial view of T1FS (**B**) sequences. STIR – Short Tau Inversion Recovery, T1FS – T1 weighted fat saturation

**Fig. 4:**
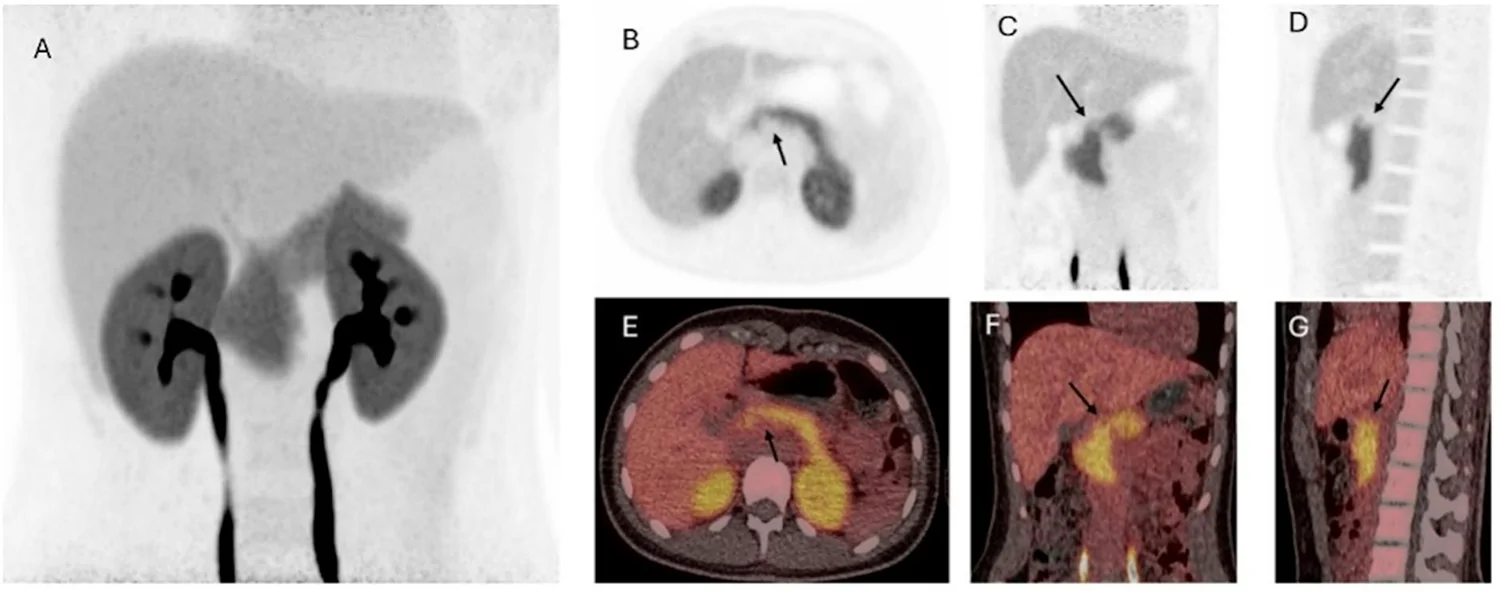
[^18^F]FDOPA PET CT images of Case Two demonstrating area of photopenia in pancreatic neck corresponding to the lesion seen on MR imaging. (**A**) MIP images does not show abnormal focal uptake. (**B**) & (**E**) – axial PET and fused PET/CT. (**C**) & (**F**) – Coronal PET and fused PET/CT. (**D**) & (**G**) – Sagittal PET and fused PET/CT, all showing photopenic area in the neck of pancreas (black arrows). Photopenia is better demonstrated on PET alone images in the top row

On transfer to our centre, a controlled fast revealed hypoglycaemia after 17 hours with inappropriately raised insulin and C-peptide levels at hypoglycaemia. Gene panel testing for congenital hyperinsulinism and MEN1 were negative. The patient was started on diazoxide with partial improvement of blood glucose levels. MR imaging (Fig. 3) demonstrated a 12 mm x 9 mm lesion in the neck of the pancreas which restricted diffusion but was occult on post-contrast sequences. The [^18^F]FDOPA PET-CT (Fig. 4) scan showed no focal increased tracer uptake on both early and delayed phase imaging. There was an area of photopenia at the site of the known lesion in the neck of the pancreas. The rest of the pancreas displayed physiological distribution of the tracer.

Ultrasound guided surgical resection of the tumour was undertaken by distal pancreatectomy at laparotomy. The insulinoma was sited at the posterior upper neck of the pancreas lying at the confluence of the inferior mesenteric and splenic veins. Histopathology confirmed an insulinoma (Grade 1, pT1). Following tumour resection, the patient was cured of hypoglycaemia and did not show recurrence at an annual review.

### Case Three

**Fig. 5.1:**
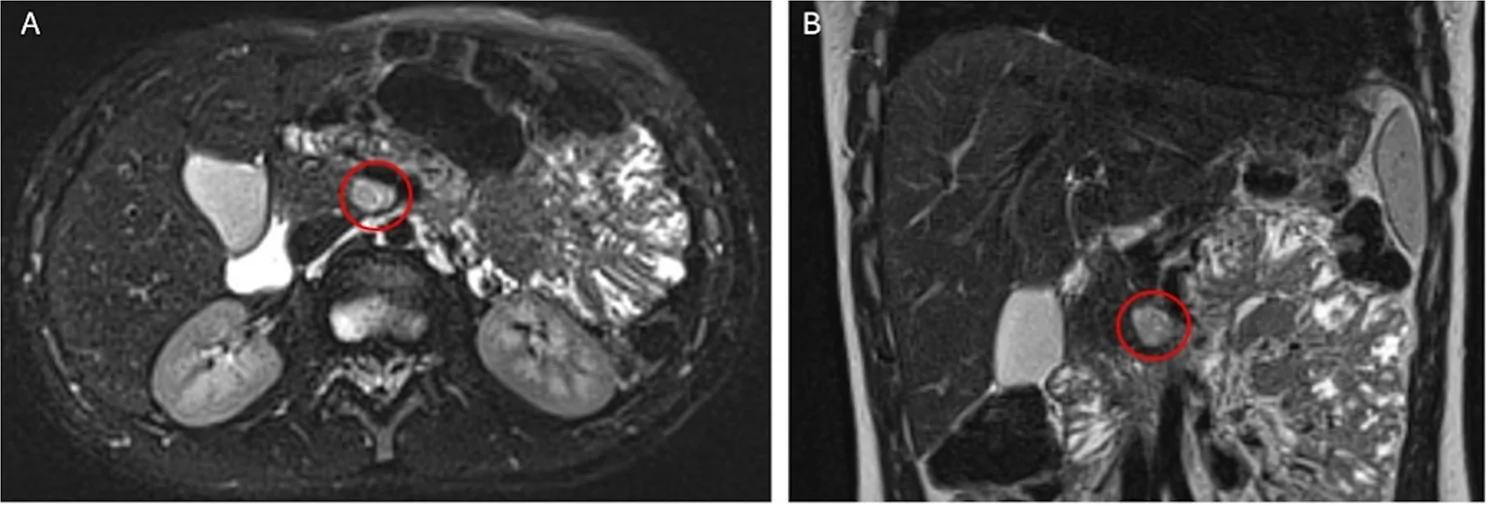
MR pancreas demonstrating hyperintense lesion (red circle) in the pancreatic uncinate process on the axial (**A**) and coronal (**B**) views of the STIR sequence. The lesion is located posterior to the superior mesenteric vessels. STIR – Short Tau Inversion Recovery

A 6 year old male presented to his local hospital with atypical seizures, not recognised to be associated with hypoglycaemia. After having been on lamotrigine for 1.5 years, he presented with a hypoglycaemic seizure. A hypoglycaemia screen confirmed biochemical evidence of excess insulin secretion. An MR scan (Fig. 5.1) in his local hospital suggested a pancreatic lesion in the uncinate process. The focus was best demonstrated on T2 and relatively hypo-intense on T1, compared to the rest of the pancreatic tissue and measured 1.4 cm x 1 cm in diameter in the axial plane.

**Fig. 5.2:**
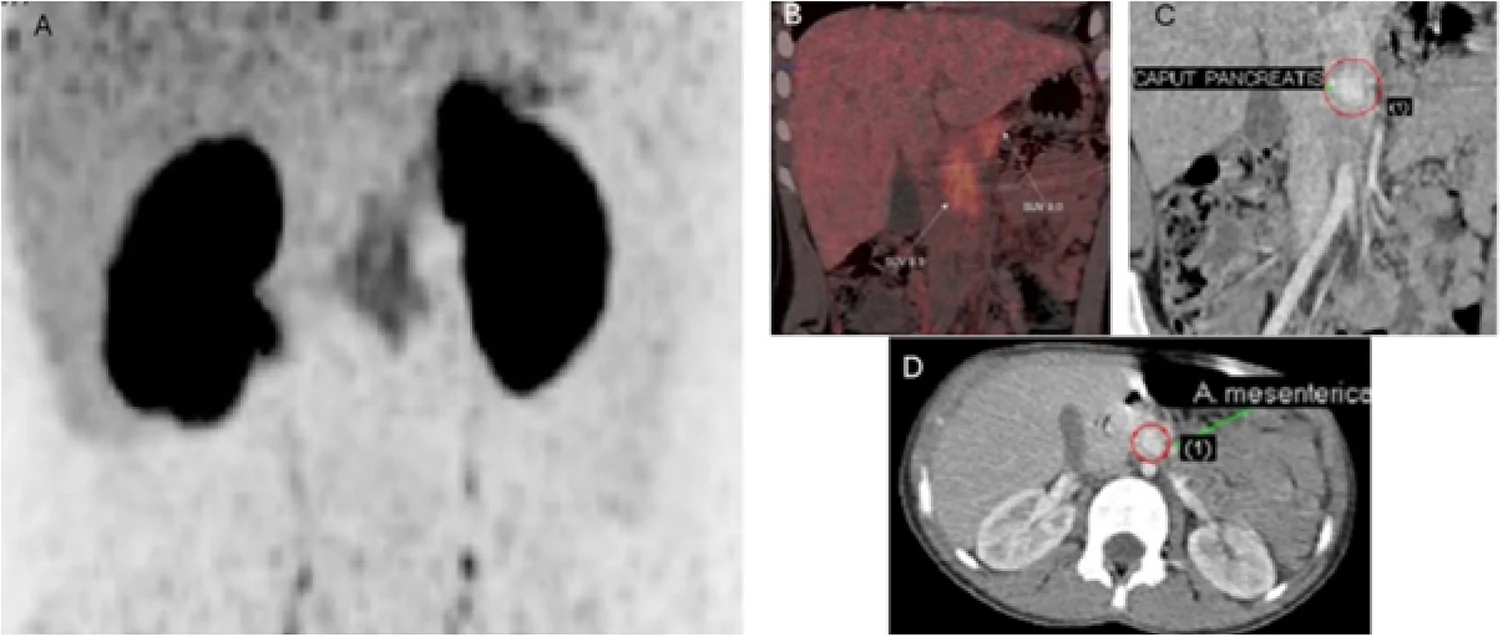
[^18^F]FDOPA PET CT images of Case Three demonstrating uptake in the uncinate process is similar to the background activity. (**A**) Maximum intensity projection (MIP) which does not show focal uptake. (**B**) Fused PET/CT showing diffuse uptake in the pancreas. (**C**) & (**D**) – Contrast CT showing enhancing lesion in the uncinate process on coronal and axial images respectively

An [^18^F]FDOPA scan (Fig. 5.2) was performed in Germany, which confirmed a well-defined lesion within the pancreas with reduction of isotope uptake. The patient underwent partial pancreatectomy that was complicated by significant post operative haemorrhage secondary to pancreatic leak. This required a jejuno-pancreatic Roux-en-Y procedure. Pancreatic histology confirmed an insulinoma; in the post-operative period, the patient was noted to carry a mutation in MEN1.

### Case Four

**Fig. 6:**
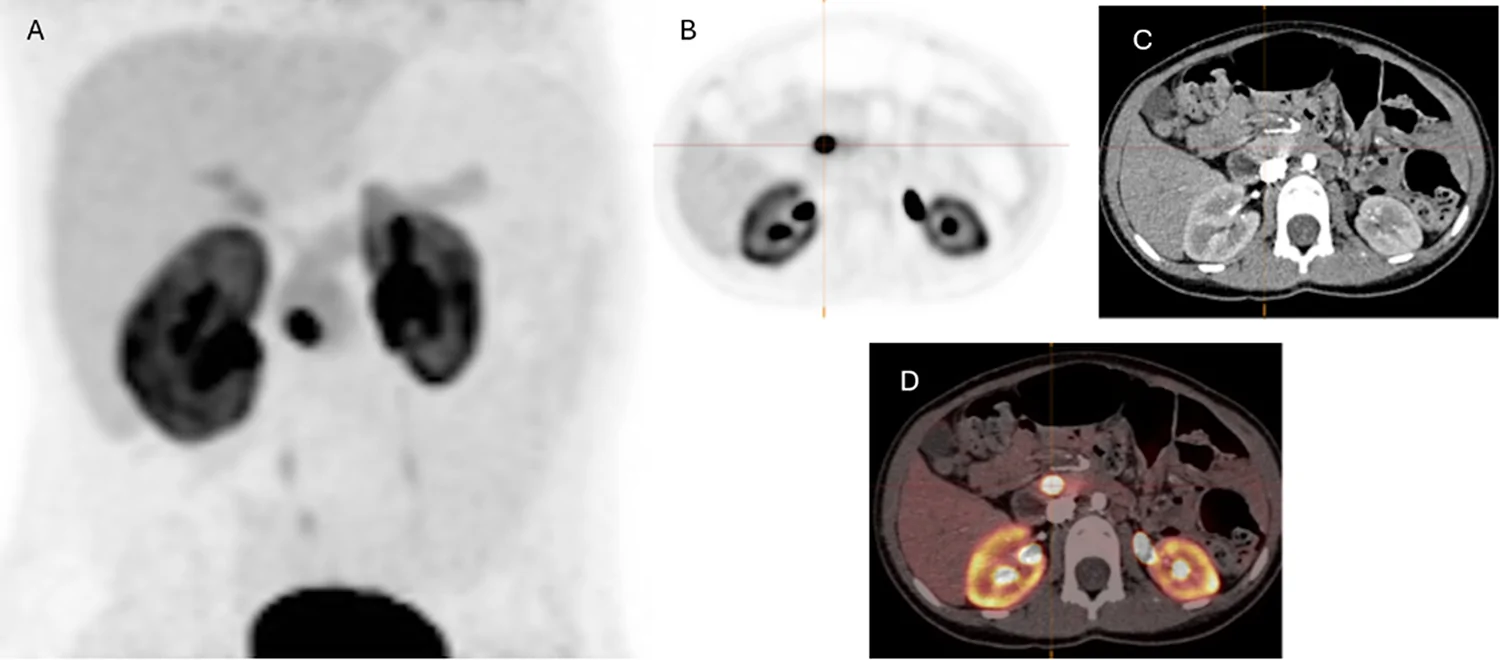
[^18^F]FDOPA PET CT images of Case Four. (**A**) – MIP images showing intense increased uptake in the uncinate process. (**B**) & (**D**) – PET and fused PET/CT axial image demonstrating focal uptake in the uncinate. (**C**) – Contrast CT showing enhancing lesion in the uncinate process

A 4 year old female presented to her local history with symptoms of hypoglycaemia; on further questioning it appeared brief symptoms of drowsiness and fatigue had been occurring for at least a year. There was biochemical evidence of hyperinsulinism on a hypoglycaemia screen. She was started on diazoxide treatment with partial response. In contrast to the previous patients in this case series, this patient had avid isotope uptake on [^18^F]FDOPA PET-CT (Fig. 6).

A histologically confirmed insulinoma was removed from the uncinate process of the pancreas with subsequent cure from hypoglycaemia. Gene panel investigations for congenital hyperinsulinism and MEN1 testing were negative.

## Discussion

It is generally known that there is increased uptake of [^18^F]FDOPA in focal lesions in infants with congenital hyperinsulinism (Hardy et al. 2007). In adult insulinomas, [^18^F]FDOPA uptake is generally increased due to high activity of the L-DOPA decarboxylase enzyme (Imperiale et al. 2015, Nakuz et al. 2018). However, our findings suggest that this pattern does not uniformly apply to paediatric insulinomas. In three of the four cases, lesions demonstrated relative photopenia, with a reduced tracer uptake compared to surrounding pancreatic tissue, rather than the expected focal avidity. Only one patient (Case Four) showed increased tracer uptake, aligning with typical adult imaging patterns. The reasoning behind this variable tracer avidity is unclear. Given that the lesions were still distinct against the rest of the pancreas, the scans were still essential for surgical planning and all four cases underwent successful tumour resection. Limitations of this case series are the small sample size and retrospective nature of data collection. Nonetheless, this series contributes valuable insight into [^18^F]FDOPA PET-CT imaging in paediatric insulinomas, given that paediatric insulinomas are incredibly rare.

## Conclusion

Our experience with four paediatric cases of insulinoma highlights photopenia, rather than increased tracer activity, was the predominant finding on [^18^F]FDOPA PET-CT imaging. Further studies are required into radiological imaging of paediatric insulinomas to further validate our findings and investigate mechanistic explanations.

## Data Availability

There are no specific datasets as this is a case series; however investigation results are available from the corresponding author upon reasonable request.
